# 2-Bromo-*N*′-[(2*Z*)-butan-2-yl­idene]-5-methoxy­benzohydrazide

**DOI:** 10.1107/S1600536809044869

**Published:** 2009-10-31

**Authors:** Jerry P. Jasinski, Ray J. Butcher, L. P. Suchitra, H. S. Yathirajan, B. Narayana

**Affiliations:** a Department of Chemistry, Keene State College, 229 Main Street, Keene, NH 03435-2001, USA; b Department of Chemistry, Howard University, 525 College Street NW, Washington, DC 20059, USA; c Department of Studies in Chemistry, University of Mysore, Manasagangotri, Mysore 570 006, India; d Department of Studies in Chemistry, Mangalore University, Mangalagangotri 574 199, India

## Abstract

In the title compound, C_12_H_15_BrN_2_O_2_, the dihedral angle between the benzene ring and the mean plane of the amide grouping is 77.7 (8)°. In the crystal, inversion dimers linked by pairs of N—H⋯O hydrogen bonds occur, and the packing is further supported by C—H⋯O and C—H⋯Br inter­actions and weak π–π ring stacking inter­actions.

## Related literature

Hydrazides and their corresponding Schiff bases are useful precursors in the synthesis of several heterocyclic systems, see: Narayana *et al.* (2005 ▶; 2005*a* ▶). For the biological activity of substituted hydrazides, see: Cajocorius *et al.* (1977 ▶). Hydrazides are inter­mediates in the production of many pharmaceutically important compounds, see: Liu *et al.* (2006 ▶). For related structures, see: Butcher *et al.* (2007 ▶); Hou (2009 ▶); Li & Ban (2009 ▶); Sarojini *et al.* (2007*a* ▶,*b* ▶,*c* ▶,*d* ▶). For the MOPAC AM1 calculations, see: Schmidt & Polik (2007 ▶).
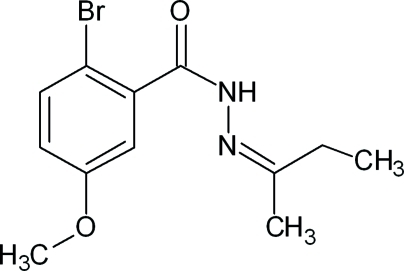


## Experimental

### 

#### Crystal data

C_12_H_15_BrN_2_O_2_
*M* *_r_* = 299.17
Monoclinic, 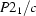

*a* = 8.0942 (1) Å
*b* = 14.2475 (2) Å
*c* = 11.2974 (2) Å
β = 91.1519 (13)°
*V* = 1302.58 (3) Å^3^
*Z* = 4
Cu *K*α radiation
μ = 4.25 mm^−1^
*T* = 200 K
0.56 × 0.47 × 0.35 mm

#### Data collection

Oxford Diffraction Gemini R CCD diffractometer
Absorption correction: multi-scan (*CrysAlis RED*; Oxford Diffraction, 2007 ▶) *T*_min_ = 0.452, *T*_max_ = 1.000
7962 measured reflections
2577 independent reflections
2484 reflections with *I* > 2σ(*I*)
*R*_int_ = 0.023

#### Refinement

*R*[*F* ^2^ > 2σ(*F* ^2^)] = 0.044
*wR*(*F* ^2^) = 0.122
*S* = 1.07
2577 reflections
157 parameters
H-atom parameters constrained
Δρ_max_ = 0.73 e Å^−3^
Δρ_min_ = −1.07 e Å^−3^

### 

Data collection: *CrysAlis Pro* (Oxford Diffraction, 2007 ▶); cell refinement: *CrysAlis Pro*; data reduction: *CrysAlis Pro*; program(s) used to solve structure: *SHELXS97* (Sheldrick, 2008 ▶); program(s) used to refine structure: *SHELXL97* (Sheldrick, 2008 ▶); molecular graphics: *SHELXTL* (Sheldrick, 2008 ▶); software used to prepare material for publication: *SHELXTL*.

## Supplementary Material

Crystal structure: contains datablocks global, I. DOI: 10.1107/S1600536809044869/ds2010sup1.cif

Structure factors: contains datablocks I. DOI: 10.1107/S1600536809044869/ds2010Isup2.hkl

Additional supplementary materials:  crystallographic information; 3D view; checkCIF report

## Figures and Tables

**Table 1:** Hydrogen-bond geometry (Å, °)

*D*—H⋯*A*	*D*—H	H⋯*A*	*D*⋯*A*	*D*—H⋯*A*
C7—H7*B*⋯O2^i^	0.98	2.60	3.561 (4)	166
C10—H10*A*⋯Br^ii^	0.98	3.07	3.949 (5)	151
C10—H10*A*⋯O2^iii^	0.98	2.55	3.231 (4)	127
C11—H11*A*⋯O1^iv^	0.99	2.55	3.373 (4)	141
N1—H1*A*⋯O2^iii^	0.88	2.07	2.932 (3)	165

Symmetry codes: (i) 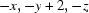; (ii) 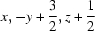; (iii) 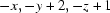; (iv) 
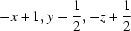
.

**Fig. 1.**
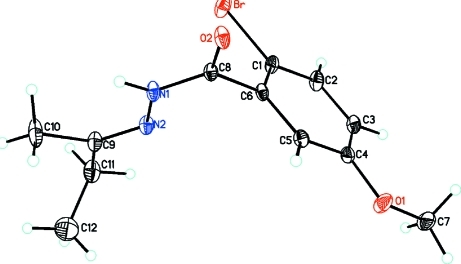
Molecular structure of C12H15BrN2O2 showing atom labeling scheme and 50% probability displacement ellipsoids.

**Fig. 2.**
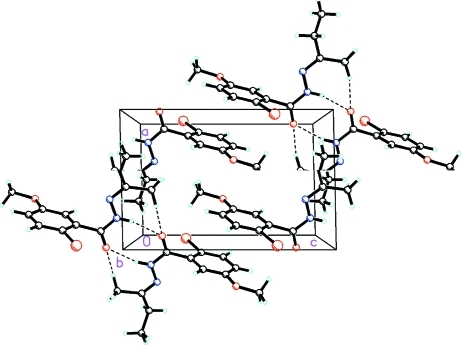
Packing diagram of the title compound, (I), viewed down the b axis. Dashed lines indicate intermediate intermolecular N—H···O and C—H···O interactions which produces a network of infinite O—H···O—H···O—H chains arranged diagonally along the (101) plane of the unit cell.

## supplementary crystallographic information

### Comment

Hydrazides and the corresponding Schiff bases are useful precursors in the synthesis of several heterocyclic systems (Narayana *et al.* 2005; 2005*a*). Some substituted hydrazides are reported to exhibit carcinostatic activity against several types of tumors (Cajocorius *et al.* 1977) and also possess antimicrobial activity. It is also used as an intermediate in many pharmaceutically important compounds (Liu *et al.* 2006). In continuation with our studies on the structures of hydrazides and their Schiff bases (Sarojini *et al.* 2007*a*, 2007*b*, 2007*c*, 2007d; Butcher *et al.* 2007) a new Schiff base, (I), C_12_H_1_5BrN_2_O_2_, has been synthesized and its crystal structure is now reported.

In the title compound, C_12_H_1_5BrN_2_O_2_, (Fig. 1), the 2-bromo and 5-methoxy groups are in the plane of the benzene ring. The dihedral angle between the mean planes of the carbonyl group (–C6—C8(O2)—N1—N2-) and benzene ring is 77.7 (8)°. The C1—C6—C8—O2 and C1—C6—C8—N1 torsion angles (-101.1 (3)° & -103.7 (3)°) support this observation. Crystal packing is supported by a collection of intermediate N1—H1A—O2 (*-x,-y + 2,-z + 1*) intermolecular interactions (see Table 1) which produces a cooperative network of infinite O—H···O—H···O—H chains arranged diagonally along the (101) plane of the unit cell (Fig. 2). In addition, weak intermolecular C10—H10A···O2 (*-x,-y + 2,-z + 1*), C11—H11A···O1 (*-x + 1, y - 1/2,-z + 1/2*), C7—H7B···O2 (*-x,-y + 2,-z*) and C10—H10A···Br (*x,-y + 3/2,z1/2*) interactions (Table 1) along with *Cg*1···*Cg*1 π-π ring stacking interactions at 3.869 (1)Å (*2 - x,1 - y,1 - z*; slippage = 1.43 (2) Å, where *Cg*1 = C1—C6), collectively, slightly influence crystal packing in this crystalline environment.

After a MOPAC AM1 computational calculation (Schmidt, 2007), the dihedral angle between the mean planes of the carbonyl group (–C6—C8(O2)—N1—N2-) and benzene ring becomes 84.0 (8)°, significantly greater that the 77.7 (8)° seen in the crystal. This supports the observation of a collective action of the intermediate and weak hydrogen bond interactions along with weak intermolecular π-π stacking interactions which influence crystal packing stability.

### Experimental

A mixture of 2-bromo-5-methoxybenzohydrazide (2.45 g, 0.01 mol) and ethyl methyl ketone(1.44 g, 0.02 mol) in 20 ml of ethanol containing a drop of dilute sulfuric acid was refluxed for about 2 h (Scheme 2). On cooling, the solid separated was filtered and recrystallized from ethyl methyl ketone. M.P.: 385 K. Analysis for C_12_H_15_BrN_2_O_2_: Found (Calculated): C: 48.14 (48.18); H: 5.02 (5.05%); N: 9.31 (9.36%).

### Refinement

All of the H atoms were placed in their calculated positions and then refined using the riding model with N—H = 0.88, C—H = 0.95–0.99 Å, and with *U*_iso_(H) = 1.2–1.5 *U*_eq_(C,*N*).

### Crystal data

**Table:**

C_12_H_15_BrN_2_O_2_	*F*(000) = 608
*M**_r_* = 299.17	*D*_x_ = 1.526 Mg m^−^^3^
Monoclinic, *P*2_1_/*c*	Cu *K*α radiation, λ = 1.54184 Å
Hall symbol: -P 2ybc	Cell parameters from 8517 reflections
*a* = 8.0942 (1) Å	θ = 5.0–73.4°
*b* = 14.2475 (2) Å	µ = 4.25 mm^−^^1^
*c* = 11.2974 (2) Å	*T* = 200 K
β = 91.1519 (13)°	Chunk, colorless
*V* = 1302.58 (3) Å^3^	0.56 × 0.47 × 0.35 mm
*Z* = 4	

### Data collection

**Table:**

Oxford Diffraction Gemini R CCD diffractometer	2577 independent reflections
Radiation source: fine-focus sealed tube	2484 reflections with *I* > 2σ(*I*)
graphite	*R*_int_ = 0.023
Detector resolution: 10.5081 pixels mm^-1^	θ_max_ = 73.6°, θ_min_ = 5.0°
φ and ω scans	*h* = −10→9
Absorption correction: multi-scan (*CrysAlis RED*; Oxford Diffraction, 2007)	*k* = −16→17
*T*_min_ = 0.452, *T*_max_ = 1.000	*l* = −9→13
7962 measured reflections	

### Refinement

**Table:**

Refinement on *F*^2^	Primary atom site location: structure-invariant direct methods
Least-squares matrix: full	Secondary atom site location: difference Fourier map
*R*[*F*^2^ > 2σ(*F*^2^)] = 0.044	Hydrogen site location: inferred from neighbouring sites
*wR*(*F*^2^) = 0.122	H-atom parameters constrained
*S* = 1.07	*w* = 1/[σ^2^(*F*_o_^2^) + (0.0673*P*)^2^ + 1.7115*P*] where *P* = (*F*_o_^2^ + 2*F*_c_^2^)/3
2577 reflections	(Δ/σ)_max_ < 0.001
157 parameters	Δρ_max_ = 0.73 e Å^−^^3^
0 restraints	Δρ_min_ = −1.07 e Å^−^^3^

### Special details

**Table:**

Geometry. All e.s.d.'s (except the e.s.d. in the dihedral angle between two l.s. planes) are estimated using the full covariance matrix. The cell e.s.d.'s are taken into account individually in the estimation of e.s.d.'s in distances, angles and torsion angles; correlations between e.s.d.'s in cell parameters are only used when they are defined by crystal symmetry. An approximate (isotropic) treatment of cell e.s.d.'s is used for estimating e.s.d.'s involving l.s. planes.
Refinement. Refinement of *F*^2^ against ALL reflections. The weighted *R*-factor *wR* and goodness of fit *S* are based on *F*^2^, conventional *R*-factors *R* are based on *F*, with *F* set to zero for negative *F*^2^. The threshold expression of *F*^2^ > σ(*F*^2^) is used only for calculating *R*-factors(gt) *etc*. and is not relevant to the choice of reflections for refinement. *R*-factors based on *F*^2^ are statistically about twice as large as those based on *F*, and *R*- factors based on ALL data will be even larger.

### Fractional atomic coordinates and isotropic or equivalent isotropic displacement parameters (Å^2^)

**Table:**

	*x*	*y*	*z*	*U*_iso_*/*U*_eq_	
Br	−0.00062 (5)	0.75926 (3)	0.23886 (3)	0.05362 (18)	
O1	0.3113 (3)	1.07458 (17)	−0.03054 (18)	0.0478 (6)	
O2	−0.0433 (2)	1.01275 (15)	0.35066 (16)	0.0358 (5)	
N1	0.1775 (3)	0.93324 (16)	0.42004 (18)	0.0306 (5)	
H1A	0.1550	0.9465	0.4941	0.037*	
N2	0.3148 (3)	0.87860 (17)	0.39363 (19)	0.0318 (5)	
C1	0.0954 (3)	0.85866 (18)	0.1528 (2)	0.0305 (5)	
C2	0.1316 (4)	0.8445 (2)	0.0351 (2)	0.0361 (6)	
H2A	0.1073	0.7858	−0.0012	0.043*	
C3	0.2033 (3)	0.9154 (2)	−0.0303 (2)	0.0316 (6)	
H3A	0.2285	0.9056	−0.1111	0.038*	
C4	0.2379 (3)	1.00067 (19)	0.0234 (2)	0.0294 (5)	
C5	0.1981 (3)	1.01479 (18)	0.1415 (2)	0.0285 (5)	
H5A	0.2195	1.0739	0.1775	0.034*	
C6	0.1282 (3)	0.94403 (17)	0.2066 (2)	0.0244 (5)	
C7	0.3619 (4)	1.0618 (3)	−0.1501 (3)	0.0525 (9)	
H7A	0.4202	1.1181	−0.1766	0.079*	
H7B	0.2644	1.0512	−0.2012	0.079*	
H7C	0.4357	1.0075	−0.1544	0.079*	
C8	0.0802 (3)	0.96538 (18)	0.3318 (2)	0.0258 (5)	
C9	0.4048 (3)	0.8504 (2)	0.4794 (2)	0.0357 (6)	
C10	0.3829 (5)	0.8738 (3)	0.6083 (3)	0.0624 (12)	
H10A	0.2676	0.8632	0.6295	0.094*	
H10B	0.4118	0.9397	0.6221	0.094*	
H10C	0.4551	0.8336	0.6570	0.094*	
C11	0.5478 (4)	0.7880 (3)	0.4479 (3)	0.0485 (8)	
H11A	0.5308	0.7253	0.4835	0.058*	
H11B	0.5486	0.7800	0.3609	0.058*	
C12	0.7109 (5)	0.8246 (4)	0.4882 (5)	0.0764 (13)	
H12A	0.7969	0.7786	0.4702	0.115*	
H12B	0.7097	0.8357	0.5738	0.115*	
H12C	0.7339	0.8837	0.4472	0.115*	

### Atomic displacement parameters (Å^2^)

**Table:**

	*U*^11^	*U*^22^	*U*^33^	*U*^12^	*U*^13^	*U*^23^
Br	0.0949 (4)	0.0345 (2)	0.0315 (2)	−0.01786 (16)	0.00182 (18)	0.00284 (12)
O1	0.0666 (14)	0.0508 (13)	0.0261 (11)	−0.0214 (11)	0.0079 (9)	−0.0005 (9)
O2	0.0396 (10)	0.0467 (12)	0.0210 (9)	0.0198 (8)	−0.0009 (7)	−0.0063 (8)
N1	0.0373 (11)	0.0386 (12)	0.0159 (10)	0.0145 (9)	0.0007 (8)	−0.0042 (8)
N2	0.0371 (11)	0.0346 (12)	0.0238 (11)	0.0125 (9)	0.0026 (9)	−0.0023 (9)
C1	0.0452 (14)	0.0245 (12)	0.0219 (12)	0.0002 (10)	0.0004 (10)	−0.0003 (10)
C2	0.0576 (17)	0.0292 (13)	0.0212 (13)	0.0033 (12)	−0.0032 (11)	−0.0081 (10)
C3	0.0397 (13)	0.0395 (15)	0.0157 (11)	0.0066 (11)	0.0019 (9)	−0.0066 (10)
C4	0.0322 (12)	0.0352 (14)	0.0206 (12)	−0.0005 (10)	−0.0021 (10)	−0.0007 (10)
C5	0.0342 (12)	0.0283 (12)	0.0230 (12)	0.0015 (10)	−0.0020 (9)	−0.0070 (10)
C6	0.0281 (11)	0.0275 (12)	0.0175 (11)	0.0092 (9)	−0.0018 (8)	−0.0033 (9)
C7	0.0564 (19)	0.076 (2)	0.0250 (15)	−0.0218 (17)	0.0066 (13)	0.0033 (15)
C8	0.0327 (12)	0.0255 (12)	0.0192 (11)	0.0047 (9)	−0.0004 (9)	−0.0039 (9)
C9	0.0391 (14)	0.0418 (15)	0.0263 (13)	0.0132 (12)	0.0014 (10)	0.0018 (11)
C10	0.060 (2)	0.104 (3)	0.0233 (15)	0.040 (2)	−0.0041 (14)	0.0002 (17)
C11	0.0513 (18)	0.0549 (19)	0.0393 (17)	0.0250 (15)	0.0012 (13)	0.0036 (15)
C12	0.050 (2)	0.100 (4)	0.079 (3)	0.016 (2)	0.004 (2)	0.004 (3)

### Geometric parameters (Å, °)

**Table:**

Br—C1	1.894 (3)	C5—H5A	0.9500
O1—C4	1.359 (3)	C6—C8	1.506 (3)
O1—C7	1.431 (4)	C7—H7A	0.9800
O2—C8	1.228 (3)	C7—H7B	0.9800
N1—C8	1.338 (3)	C7—H7C	0.9800
N1—N2	1.394 (3)	C9—C10	1.507 (4)
N1—H1A	0.8800	C9—C11	1.508 (4)
N2—C9	1.266 (4)	C10—H10A	0.9800
C1—C2	1.382 (4)	C10—H10B	0.9800
C1—C6	1.383 (3)	C10—H10C	0.9800
C2—C3	1.386 (4)	C11—C12	1.482 (6)
C2—H2A	0.9500	C11—H11A	0.9900
C3—C4	1.383 (4)	C11—H11B	0.9900
C3—H3A	0.9500	C12—H12A	0.9800
C4—C5	1.393 (4)	C12—H12B	0.9800
C5—C6	1.376 (4)	C12—H12C	0.9800
			
C4—O1—C7	117.4 (2)	H7A—C7—H7C	109.5
C8—N1—N2	119.4 (2)	H7B—C7—H7C	109.5
C8—N1—H1A	120.3	O2—C8—N1	121.9 (2)
N2—N1—H1A	120.3	O2—C8—C6	120.0 (2)
C9—N2—N1	117.5 (2)	N1—C8—C6	118.2 (2)
C2—C1—C6	120.6 (2)	N2—C9—C10	126.3 (3)
C2—C1—Br	118.8 (2)	N2—C9—C11	116.0 (3)
C6—C1—Br	120.59 (19)	C10—C9—C11	117.6 (3)
C1—C2—C3	120.3 (2)	C9—C10—H10A	109.5
C1—C2—H2A	119.9	C9—C10—H10B	109.5
C3—C2—H2A	119.9	H10A—C10—H10B	109.5
C4—C3—C2	119.4 (2)	C9—C10—H10C	109.5
C4—C3—H3A	120.3	H10A—C10—H10C	109.5
C2—C3—H3A	120.3	H10B—C10—H10C	109.5
O1—C4—C3	124.8 (2)	C12—C11—C9	113.8 (3)
O1—C4—C5	115.4 (2)	C12—C11—H11A	108.8
C3—C4—C5	119.8 (2)	C9—C11—H11A	108.8
C6—C5—C4	120.8 (2)	C12—C11—H11B	108.8
C6—C5—H5A	119.6	C9—C11—H11B	108.8
C4—C5—H5A	119.6	H11A—C11—H11B	107.7
C5—C6—C1	119.1 (2)	C11—C12—H12A	109.5
C5—C6—C8	118.1 (2)	C11—C12—H12B	109.5
C1—C6—C8	122.7 (2)	H12A—C12—H12B	109.5
O1—C7—H7A	109.5	C11—C12—H12C	109.5
O1—C7—H7B	109.5	H12A—C12—H12C	109.5
H7A—C7—H7B	109.5	H12B—C12—H12C	109.5
O1—C7—H7C	109.5		
			
C8—N1—N2—C9	179.2 (3)	Br—C1—C6—C5	179.97 (19)
C6—C1—C2—C3	0.8 (4)	C2—C1—C6—C8	175.6 (2)
Br—C1—C2—C3	−179.4 (2)	Br—C1—C6—C8	−4.2 (3)
C1—C2—C3—C4	−0.2 (4)	N2—N1—C8—O2	179.0 (3)
C7—O1—C4—C3	−2.5 (4)	N2—N1—C8—C6	−2.5 (4)
C7—O1—C4—C5	177.0 (3)	C5—C6—C8—O2	74.8 (3)
C2—C3—C4—O1	178.4 (3)	C1—C6—C8—O2	−101.1 (3)
C2—C3—C4—C5	−1.0 (4)	C5—C6—C8—N1	−103.7 (3)
O1—C4—C5—C6	−177.9 (2)	C1—C6—C8—N1	80.4 (3)
C3—C4—C5—C6	1.6 (4)	N1—N2—C9—C10	−3.0 (5)
C4—C5—C6—C1	−0.9 (4)	N1—N2—C9—C11	177.4 (3)
C4—C5—C6—C8	−177.0 (2)	N2—C9—C11—C12	122.5 (4)
C2—C1—C6—C5	−0.3 (4)	C10—C9—C11—C12	−57.1 (5)

### Hydrogen-bond geometry (Å, °)

**Table:**

*D*—H···*A*	*D*—H	H···*A*	*D*···*A*	*D*—H···*A*
C7—H7B···O2^i^	0.98	2.60	3.561 (4)	166
C10—H10A···Br^ii^	0.98	3.07	3.949 (5)	151
C10—H10A···O2^iii^	0.98	2.55	3.231 (4)	127
C11—H11A···O1^iv^	0.99	2.55	3.373 (4)	141
N1—H1A···O2^iii^	0.88	2.07	2.932 (3)	165

Symmetry codes: (i) −*x*, −*y*+2, −*z*; (ii) *x*, −*y*+3/2, *z*+1/2; (iii) −*x*, −*y*+2, −*z*+1; (iv) −*x*+1, *y*−1/2, −*z*+1/2.
